# Improved EAV-Based Algorithm for Decision Rules Construction

**DOI:** 10.3390/e25010091

**Published:** 2023-01-02

**Authors:** Krzysztof Żabiński, Beata Zielosko

**Affiliations:** Institute of Computer Science, Faculty of Science and Technology, University of Silesia in Katowice, Będzińska 39, 41-200 Sosnowiec, Poland

**Keywords:** decision rules, length, support, greedy heuristics, feature selection, rough sets

## Abstract

In this article, we present a modification of the algorithm based on EAV (entity–attribute–value) model, for induction of decision rules, utilizing novel approach for attribute ranking. The selection of attributes used as premises of decision rules, is an important stage of the process of rules induction. In the presented approach, this task is realized using ranking of attributes based on standard deviation of attributes’ values per decision classes, which is considered as a distinguishability level. The presented approach allows to work not only with numerical values of attributes but also with categorical ones. For this purpose, an additional step of data transformation into a matrix format has been proposed. It allows to transform data table into a binary one with proper equivalents of categorical values of attributes and ensures independence of the influence of the attribute selection function from the data type of variables. The motivation for the proposed method is the development of an algorithm which allows to construct rules close to optimal ones in terms of length, while maintaining enough good classification quality. The experiments presented in the paper have been performed on data sets from UCI ML Repository, comparing results of the proposed approach with three selected greedy heuristics for induction of decision rules, taking into consideration classification accuracy and length and support of constructed rules. The obtained results show that for the most part of datasests, the average length of rules obtained for 80% of best attributes from the ranking is very close to values obtained for the whole set of attributes. In case of classification accuracy, for 50% of considered datasets, results obtained for 80% of best attributes from the ranking are higher or the same as results obtained for the whole set of attributes.

## 1. Introduction

Decision rules are one of popular and well-known form of data representation. They are also often used in the classifier building process. Generally, it can be said that the process of induction of decision rules may have two perspectives [1]: knowledge representation and classification.

One of the main purposes of knowledge representation is to discover patterns or anomalies hidden in the data. The patterns are presented in the form of decision rules that map dependencies between the values of conditional attributes and the label of the decision class. Taking into account this perspective of rule induction, there exists variety of rules’ quality measures that are related to human perception. These are, among others number of induced rules, their length and support [2,3].

The purpose of rule-based classifier is to assign a decision class label to a new object based on the attributes values’ describing that object. One of the popular measure of rule quality from this perspective belonging to the domain of supervised learning, is the classification error. It is a percentage of the number of incorrectly classified examples.

There are different approaches for construction of decision rules. It is known that the form of obtained rules, for example, their number, length, depend on the algorithm used for their induction. Moreover, the set of rules which consist of a classifier ensuring a low classification error, is not always easy to understand and interpret from the point of view of knowledge representation. On the other hand, a small number of induced rules that are short and only reflect general patterns from the data, will not always ensure a good classification quality. These discrepancies mean that different rule induction approaches may be proposed, depending on the purpose of their application and mentioned two perspectives of rule induction, i.e., classification and knowledge representation, which do not coincide often.

In the paper, an approach that allows induction of decision rules, taking into account both the knowledge representation and classification perspective is presented. The proposed algorithm is based on the idea of an extension of the dynamic programming approach for optimization of decision rules relative to length and partitioning table into subtables.

Unfortunately, for large data sets, i.e., with a large number of attributes with many different values, the time for obtaining an optimal solution may be relatively long, which motivated authors to develop the presented method. Moreover, the problem of minimization of length of decision rules is NP-hard [4,5] and the most part of approaches for decision rules construction, with the exception of brute force, Boolean reasoning, extension of dynamic programming, Apriori algorithm, cannot guarantee the construction of optimal rules, i.e., rules with minimum length. Exact algorithms for construction of decision rules with minimum length have very often exponential computational complexity. Thus, for large datasets the rule generation time can be significant. However, often results close to optimal ones are enough for given application. Taking into account above facts, some heuristic which allows to obtain rules close to optimal from the point of view of length and with relatively good accuracy of classification was presented. The proposed algorithm is an extension and modification of the approach presented in [6]. To ensure the possibility of working with categorical values of attributes, and the independence of the attribute selection function from the data type, the data preparation stage was introduced. It consist of transforming data set into a matrix form and allows to work with binary data table where each attribute value has the same weight and numerical values are assigned automatically. This step is important from the point of view of attribute selection process performed during rule construction phase. An other element of the proposed approach is transformation data table into EAV (attribute–entity–value) form which is convenient for processing large amounts of data.

The methods and approaches for choosing of the attributes that consist of rules’ premises can be wrapped in the rule induction algorithms or can be performed immediately preceding the rule induction step. An example of the latter approach is rule construction based on reducts [7]. However, in both cases different measures, such as based on similarity, entropy, dependency, distance or statistical characteristics are employed and used for attributes evaluation. It is also possible that based on selected set of features their ranking is constructed. It allows to indicate importance of variables. In the paper, the method for selection of attributes directly precedes the rule induction step. It takes into account an influence of features’ values into class labels and it is based on standard deviation of attributes values per decision classes. Obtained values of standard deviation function are used for creation of ranking of variables and user decides what percentage of attributes with highest position in the ranking is taken into account during rule construction phase.

Decision rules induced by presented algorithm were compared with three selected heuristics. The choice of these heuristics follows from the fact that they allow to obtain rules close to optimal ones in terms of length and support. In [8] the experimental results showed that the average relative difference between length of rules constructed by the best heuristic and minimum length of rules is at most 4%, similar situation was observed in case of support.

The paper consists of five sections. Section 2 is devoted to approaches and methods for attribute selection during process of induction of decision rules. The main stages of the proposed algorithm are presented in Section 3. Section 4 contains short description of three selected heuristics for induction of decision rules. Experimental results concerning analysis of obtained sets of rules from the point of view of knowledge representation and classification, and comparison with selected heuristics are included in Section 5. Conclusions and future plans are given in Section 6.

## 2. Selection of Attributes for Rule Construction

The attribute selection process, in general, leads to the selection of a certain subset of originally available features in order to accomplish a specific task, which is, e.g., creation a model for classification purposes [9]. It also allows for removal redundant or irrelevant variables from a set of all attributes. The feature selection stage is not only an important element of data preprocessing, it plays a key role during induction of decision rules. The obtained results impact on the knowledge representation perspective. A smaller set of attributes is easier to check, understand and visualize, it has lower storage requirements and from the classification point of view it allows to avoid overfitting [10]. Selection of features can lead to the creation of their ranking. This approach is called feature ranking and allows to estimate relevance of attributes based on some adopted threshold. As a result, the most important variables have assigned the highest positions in the ranking, and the least relevant—the lowest positions.

There are many algorithms for selecting features. The most popular is a division of methods into filters, wrappers, and embedded [11]. Filter methods can be considered as data preprocessing tasks that are independent on the classification systems. Therefore, their advantage is speed and main drawback is what makes them fast and easily applicable in almost all kinds of problems, i.e., neglecting the real-time influence on the classification system. Wrapper methods, as opposed to filters, can be treated as feedback-based systems by examining the influence of the choice of subsets of features on the classification result. The last group, embedded methods contain a feature subset evaluation mechanism built directly into the learning algorithm. As a result, they can provide good quality solutions for specific applications where knowledge about characteristics of learning algorithm is necessary.

A decision rule can be viewed as a hypothesis that maps to a pattern in the data or a function that predicts a decision class label for a given object. From this perspective, selection of attributes is one of element of decision rule construction process. It is often performed during the rule induction algorithm work and it is an iterative step in which the attributes are selected sequentially if adopted criterion is met. It is also possible to construct rules using filter approach, e.g., based on reducts. In both cases, the chosen attribute together with the corresponding value form a rule descriptor (attribute = value pair) which constitutes a rule premise part. The attributes contained in rules determine their quality, therefore the process of variable selection and the adopted criterion plays an important role.

In the framework of rough sets theory there are many algorithms for induction of decision rules [12]. During process of rules construction different evaluation measures are used and they are based on discernibility relation, upper and lower approximations, dependency degree concept, discernibility function and prime implicants and many others [13,14]. Reduct is a popular notion in the rough sets theory [15] and is interpreted as such minimal subset of attributes that is sufficient to discern any pairs of objects with different class labels. Based on the attributes which constitutes reduct, decision rules are constructed, so they are induced from the reduced set of attributes [16,17]. The popular measures for selection of attributes during reduct construction are based on, for example, discernibility matrix [18], positive region-based dependency [19], neighbourhood information granules [20], entropy and many others [21].

Another group of methods related to algorithms for induction of decision rules is based on sequential covering approach [22,23], e.g., family of AQ algorithms, CN2, Ripper. In this framework, candidates for the elementary conditions of a rule are evaluated taking into account, for example, maximization of the number of positive examples covered by the conjunction of elementary conditions in premise part of a rule, maximization of the ratio of covered positive examples to the total number of covered examples, minimization of a rule length and others [24,25].

It should be also noted that there are many heuristics algorithms which uses different criteria based on entropy, Gini index, information gain, statististical characteristics and different their modifications [26,27,28,29,30,31,32].

In the proposed approach, selection of attributes is based on standard deviation of attributes values in the framework of decision classes, described in Section 3.

## 3. Decision Rules Construction Approach

**Figure 1:**
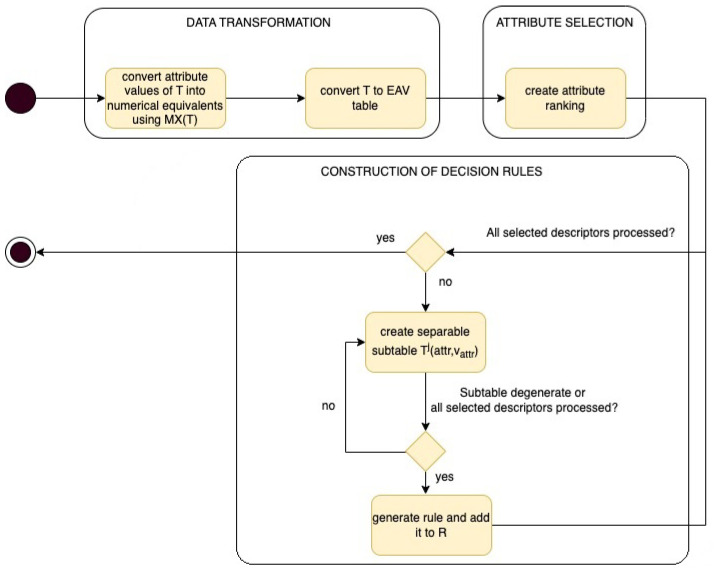
General idea of the approach for decision rules construction.

In this section, an algorithm for decision rules induction is presented. This algorithm can be considered as an extension and improvement of the algorithm based on EAV model presented in [6]. One of the important element of the considered approach is selection of attributes based on standard deviation of their values in the framework of decision classes. In order to calculate standard deviation of attributes values, categorical ones should be transformed to numerical. The modification proposed in this paper provides independence of the attribute selection function from the data type of variables and automatic assignment of numerical equivalents to categorical values, so each attribute has the same weight. This stage of the algorithm is considered as data preparation step which concerns transformation data table into matrix form [33]. Then, based on numerical form of data, EAV table [34] is created which allows to use the relational database engine to determine the standard deviation of attributes within decision classes. This step of proposed approach is presented as data transformation block on Figure 1. Employing selection of attributes based on standard deviation approach results ranking of features that indicates order and importance of attributes which are considered during process of rule construction. This stage of the approach is presented as attribute selection block on Figure 1. The third phase is indicated on Figure 1 as construction of decision rules block. The general idea of the proposed approach, expressed in the form of an activity diagram, is presented on the Figure 1 and described in detail in the following sections.

### 3.1. Data Transformation and Attribute Selection

Popular form of data representation is tabular form defined as a decision table *T* [15], T=(U,A∪{d}), where *U* is a nonempty, finite set of objects (rows), $A=\{attr_{1},\ldots ,attr_{n}\}$ is nonempty, finite set of condition attributes, $attr:U\to V_{attr}$ is a function, for any attr∈A, $V_{attr}$ is the set of values of an attribute attr. d∉A is a distinguished attribute called a decision attribute with values $V_{d}=\left\{d_{1},\ldots ,d_{|V_{d}|}\right\}$.

Data transformation stage consists of data transformation into matrix form and construction of EAV table. The first one is applied in order to facilitate statistical analysis if the attributes’ values are categorical. Such way of data preparation is known from CART (ang. classification and regression trees) approach [35] and also used for induction of binary association rules [36]. It is a tabular form where each attribute and its value from *T* is represented as a single table column. Matrix data format incorporates two attribute values only: 0 or 1. 1 represents the situation where a given attribute with its value occurs for the given object, 0 represents the situation where a given attribute with its value does not occur for the given row of *T*. Algorithm 1 presents conversion of symbolic values of attributes from data table *T* into matrix form MX(T).
**Algorithm 1** Algorithm for conversion of symbolic values of attributes into numerical equivalents.**Input:** decision table *T* with condition attributes $attr_{1},\ldots ,attr_{n}$, row $r=(v_{attr_{1}},\ldots ,v_{attr_{n}})$**Output:**MX(T)-matrix data form of *T*  AV←∅; //AV is a set of unique pairs ($attr,v_{attr}$) from *T*  **for each**
*r* of *T*
**do**    add descriptor ($attr,v_{attr}$) to AV;  **each for**  **for each** descriptor ($attr,v_{attr}$) from AV
**do**    add column to MX(T), named $av_{i}$, filled with 0’s;  **end for**  **for each**
*r* of *T*
**do**    set value to 1 for column named $av_{i}$ where a=attr and $v_{i}=v_{attr}$;  **end for**

**Figure 2:**
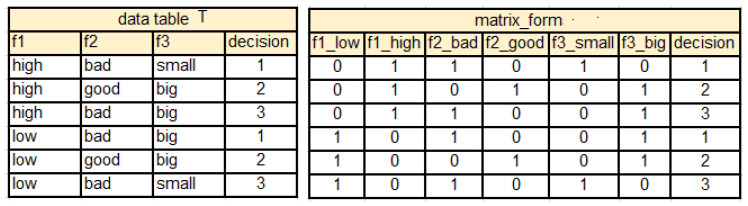
Data table *T* transformed to matrix form.

An example of data table transformed into the matrix form is presented in Figure 2.

Based on data presented in the matrix form, average values of each column of MX(T) are obtained and used for replacement of symbolic values of attributes by their numerical equivalents in the table *T*.

The next stage of data transformation concerns conversion of a decision table with numerical equivalents into EAV form. It is a tabular form where each row contains an attribute, its corresponding value, class label and the ordinal number of object to which the given attribute is assigned. The main advantage of this approach is the possibility of using a relational database engine to analyze large data sets, as it was shown in case of induction of association and decision rules [37,38].

**Figure 3:**
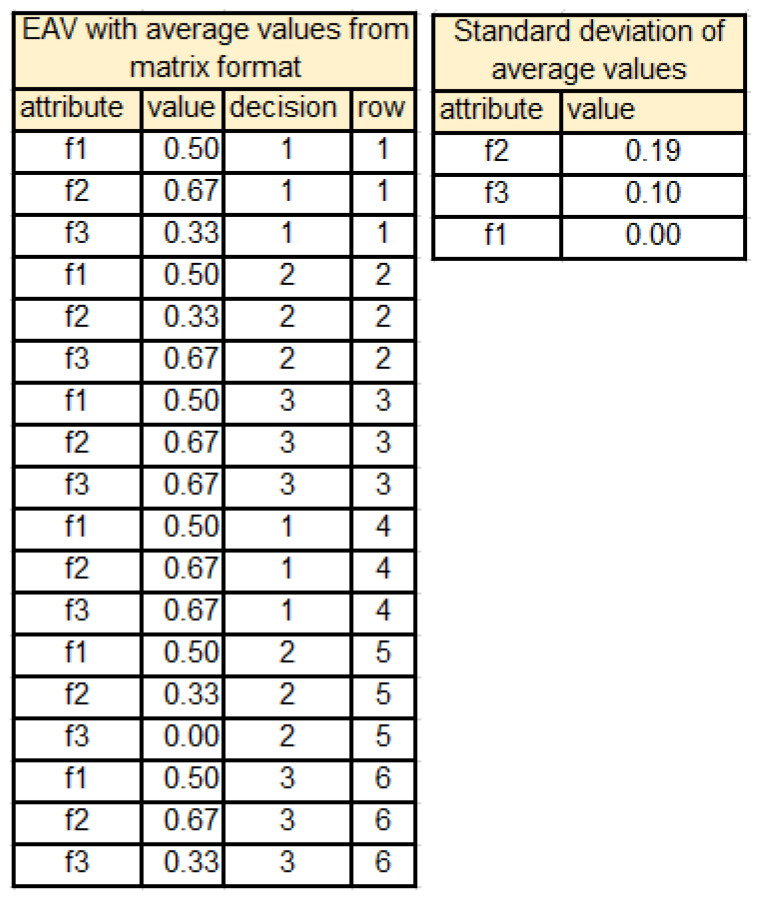
EAV table and ranking of attributes for data presented in Figure 2.

Then, calculation of standard deviation of attributes values per decision class is performed and ranking of attributes is obtained (see Figure 3).

The standard deviation of average values of attributes per decision classes has been chosen as a distinguishability level, following the intuitive idea that there is a correlation between average attribute value in a given class and the class itself. The relation is directly proportional, meaning that the highest the average standard deviation of the attribute, the biggest impact on the decision class. This intuitive approach follows the ideas of Bayesian analysis of data using Rough Bayesian model, which has been introduced in [39]. There was shown a correspondence between the main concepts of rough set theory and statistics where a hypothesis (target concept $X_{1}$) can be verified positively, negatively (in favour of the null hypothesis, which is a complement concept $X_{0}$) or undecided, under the given evidence *E*. The Rough Bayesian model is based on the idea of inverse probability analysis and Bayes factor $B_{0}^{1}$, defined as follows [39]:

$$B_{0}^{1}=\frac{Pr(E|X_{1})}{Pr(E|X_{0})}.$$

Posterior probabilities can correspond to the accuracy factor in the machine learning domain [40]. Comparison of prior and posterior knowledge allows seeing if new evidence (satisfaction of attributes’ values of objects) decreases or increases the belief in a given event, i.e., membership to a given decision class.

Let us assume that $X_{k}$ are events, then $Pr(X_{k})$ is the prior probability, $\sum_{l=0}^{|V_{d}|-1}Pr\left(X_{l}\right)=1$. It is possible that $X_{k}$ will occur, but there is no certainty for that. $Pr(X_{k}|E)$ is the posterior probability meaning $X_{k}$ can occur when the evidence associated with *E* appears, $\sum_{l=0}^{|V_{d}|-1}Pr\left(X_{l}|E\right)=1$. *E* can be considered in the framework of indiscernibility relation E∈U/B, B∈A, which provides a partition of objects *U* from decision table *T* into groups having the same values of *B*. The above-mentioned probabilities can be estimated as follows:

$$Pr\left(X_{k}\right)=\frac{|X_{k}|}{\left|U\right|},Pr\left(X_{k}|E\right)=\frac{|X_{k}\cap E|}{\left|E\right|}.$$

Obviously, the bigger value of $Pr(X_{k}|E)$ is, the higher correlation between $X_{k}$ and *E* exists. Then, using the probability density function, it is possible to visualize the influence of the posterior probability on the density range of *E*. This range can be approximated using the standard deviation of the attribute values within a given decision class. Such an approach was used in the feature selection process [41] and induction of decision rules [6,34,37].

### 3.2. Construction of Decision Rules

Based on the created ranking of attributes, it is possible to proceed to rules generation stage. In the proposed approach, user can indicate a specified number of best attributes which will be taken into consideration during the process of rules induction. On this basis, descriptors from set AV, which is a set of unique pairs ($attr,v_{attr}$) from *T*, are selected. Starting with the highest ranked attribute, a separable subtable is created. It is a subtable of the table *T* that contains only rows that have values $v_{attr_{1}},\ldots ,v_{attr_{m}}$ at the intersection with columns $attr_{i_{1}},\ldots ,attr_{i_{m}}$ and is denoted by $T^{\prime }=T\left(attr_{i_{1}},v_{attr_{1}}\right)\ldots \left(attr_{i_{m}},v_{attr_{m}}\right)$. The process of the partitioning of the table *T* into separable subtables is stopped when the considered subtable is degenerate, i.e., the same decision values are assigned to all rows or when all descriptors from AV based on the selected attributes were used. Pairs $\left(attr=v_{a}ttr\right)$ that form separable subtables $T^{\prime }$ at the bottom level corresponds to descriptors included in the premise part of decision rules. $mcd(T^{\prime })$ denotes the most common decision for rows of $T^{\prime }$. Algorithm 2 presents the algorithm for decision rules construction.
**Algorithm 2** Algorithm for induction of decision rules.**Input:** decision table *T* with numerical values of attributes, number *p* of best attributes to be taken into consideration**Output:** set of unique rules *R*  j←∅;  Q←∅;  convert *T* into EAV table;  $\forall_{attr\in A}$ calculate $STD_{attr}$ grouped by $V_{d}$ and create a ranking;  select *p* attributes from the ranking and select descriptors from AV containing selected attributes;  **while** all selected descriptors are not processed **do**    create separable subtable $T^{j}\left(attr,v_{attr}\right)$;    $Q\leftarrow Q\cup \{attr=v_{attr}\}$;    **if** $T^{j}\left(attr,v_{attr}\right)$ is degenerate OR j=p
**then**      $R\leftarrow R\cup \forall_{attr=v_{attr}\in Q}\left(attr_{i}=v_{attr_{i}}\right)\to mcd\left(T^{j}\right)$, where $mcd(T^{j})$ is the most common decision for $T^{j}$;    **else**      j=j+1;    **end if****end while**

The time and space complexity of the Algorithm 2 has been discussed in details in the previous authors’ publication [6]. The mean computational complexity is linear and only decision table specificity can lead to square complexity in the worst case scenario. Algorithm 1 is part of the whole approach for decision rule construction with minor influence on the whole complexity itself.

## 4. Selected Greedy Heuristics

Greedy algorithms are often used to solve optimization problems. This approach, in order to determine the solution at each step, makes a greedy, i.e. the most promising partial solution at a given moment.

In the paper, three greedy heuristics are presented. They are called *M*, RM and log and used for rule induction. Detailed description of these heuristics can be found in [8]. The research has shown that on average the results of the greedy algorithms, in terms of length and support of induced rules, are close to optimal ones obtained by extensions of dynamic programming approach.

In general, the pseudocode of greedy heuristics is presented by Algorithm 3. Each heuristic (*M*, RM or log) constructs a decision rule for the table *T* and a given row *r* with assigned decision $d_{k}$, $k\in \{1,\ldots ,|V_{d}|\}$. It is applied sequentially, for each row *r* of *T* and in each iteration selects an attribute $attr_{i}\in \left\{attr_{1},\cdots ,attr_{n}\right\}$ with a minimum index, fulfilling the given criterion.
**Algorithm 3** Heuristic (*M*, RM or log) for induction of decision rules.**Input:** Decision table *T* with condition attributes and row *r***Output:** Decision rule rul for *T* and given row *r*  Q←∅;  $T^{0}\left(attr,v_{attr}\right)\leftarrow T$;  **while**
$T^{j}\left(attr,v_{attr}\right)$ is not degenerate **do**    select attribute $attr_{i}$ as follows:    • heuristic *M* selects $attr_{i}$ which minimizes the value $M(attr_{i},r,d_{k})$;    • heuristic RM selects $attr_{i}$ which minimizes the value $RM(attr_{i},r,d_{k})$;    • heuristic log selects $attr_{i}$ which maximizes the value $\frac{\beta (attr_{i},r,d_{k})}{\log_{2}\left(\alpha \left(attr_{i},r,d_{k}\right)+2\right)}$;    Q←Q∪{attr};    $T^{\left(j+1\right)}\leftarrow T^{j}\left(attr,v_{attr}\right)$;    j=j+1;  **end while**  $rul\leftarrow \forall_{attr\in Q}\left(attr_{i}=v_{attr_{i}}\right)\to d_{k}$;

During the heuristics work, the following notation was used: N(T)-number of rows in the table *T*, $N(T,d_{k})$-number of rows from *T* with a given decision.

- $M\left(attr_{i},r,d_{k}\right)=M\left(T^{j},d_{k}\;\right)$$=N\left(T^{j+1}\right)-N\left(T^{j+1},d_{k}\right)$,
- $RM\left(attr_{i},r,d_{k}\right)=\left(N\left(T^{j+1}\right)-N\left(T^{j+1},d_{k}\right)\right)/N\left(T^{j+1}\right)$,
- $\alpha (attr_{i},r,$$d_{k})=N\left(T^{j},d_{k}\right)-N\left(T^{j+1},d_{k}\right)$ and $\beta \left(attr_{i},r,d_{k}\right)=M\left(T^{j},d_{k}\right)-M\left(T^{j+1},d_{k}\right).$

**Figure 4:**
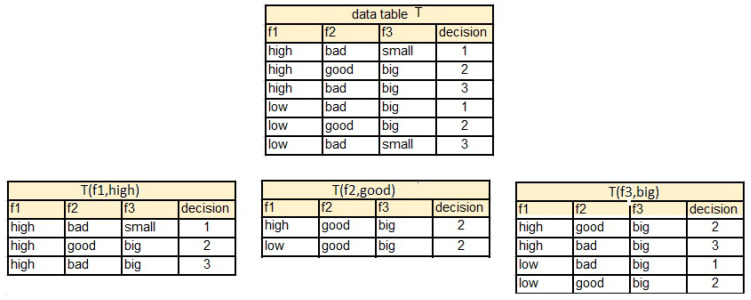
Separable subtables $T\left(f_{1},high\right),T\left(f_{2},good\right),T\left(f_{3},big\right)$ of decision table *T*.

Figure 4 presents separable subtables created based on the values of attributes assigned to the second row of data table *T*.

The selected heuristics work as follows:

- $M(f_{1},r_{2},2)=2$, $M(f_{2},r_{2},2)=0$, $M(f_{3},r_{2},2)=2$,
  $f_{2}=good\to 2$;
- $RM\left(f_{1},r_{2},2\right)=\frac{1}{3}$, $RM(f_{2},r_{2},2)=0$, $RM\left(f_{3},r_{2},2\right)=\frac{1}{2}$,
  $f_{2}=good\to 2$;
- $\alpha (f_{1},r_{2},2)=1$, $\alpha (f_{2},r_{2},2)=0$, $\alpha (f_{3},r_{2},2)=0$,
  $\beta (f_{1},r_{2},2)=2$, $\beta (f_{2},r_{2},2)=4$, $\beta (f_{3},r_{2},2)=0$,
  $f_{2}=good\to 2$;

Decision rules constructed by these heuristics for the second row from *T* are the same.

## 5. Experimental Results

Experiments have been executed on datasets from UCI Machine Learning Repository [42]. Unique valued attributes have been eliminated. Any missing values have been filled by the most common value for the given attribute. The sets taken into consideration are the following:

- balance-scale,
- breast-cancer,
- cars,
- flags,
- hayes-roth-data,
- house-votes,
- lymphography,
- tic-tac-toe.

The aim of the experiments is to compare the proposed algorithm with the selected heuristics. The study was performed from the point of view of knowledge representation taking into account length and support of constructed rules and from the point of view of classification accuracy. Length of the rule is defined as number of descriptors in the premise part of the rule. Support of the rule is the number of rows from *T* which matching conditions and the decision of a given rule. Classification accuracy is defined as the number of properly classified rows from the test part of *T*, divided by the number of all rows from the test part of *T*.

The algorithms have been implemented in Java 17 and Spring Boot framework and experiments have been executed with Macbook Pro: Intel i7-9750H CPU, 16 GB of RAM memory, macOS Monterey 12.2.1 operating system.

### 5.1. Comparison from the Point of Data Representation

From the point of view of data representation, two quality measures have been compared: rule length and rule support. Table 1, Table 2 and Table 3 present minimal, average and maximal length and support of rules obtained by proposed algorithm taking into account 100%, 80% and 60% of best attributes from the ranking.

**Figure 5:**
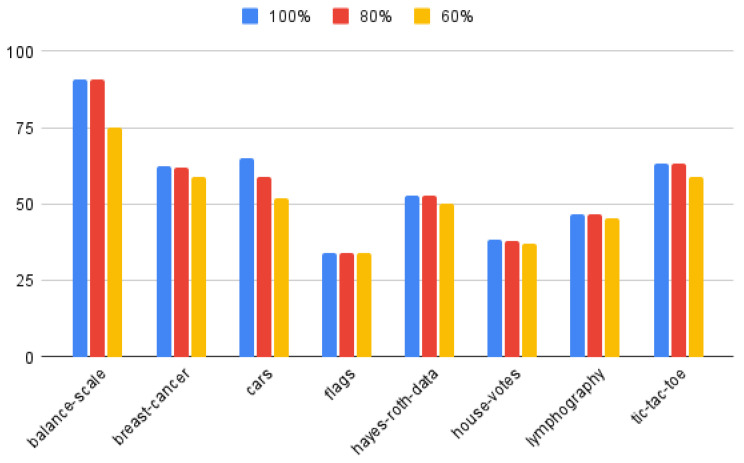
The average length of rules relative to number of attributes in given dataset, obtained for 100%, 80% and 60% of best attributes from the ranking.

Figure 5 presents, the average length of rules relative to number of attributes, obtained for 100%, 80% and 60% of best attributes from the ranking, for considered datasets. It is possible to see that for most of the datasets, with the exceptions of breast-cancer and cars, the average length of rules obtained for 80% of best attributes from the ranking is very close to results obtained for the whole set of attributes. In case of average support the best results, were obtained for datasets cars and house-votes. The function that determines the choice of attributes during decision rule construction is the standard deviation of attribute values within decision classes. Thus, the distribution of such values has an impact on the obtained results.

Table 4, Table 5 and Table 6 present minimal, average and maximal length and support of rules obtained by heuristics *M*, RM and log.

**Figure 6:**
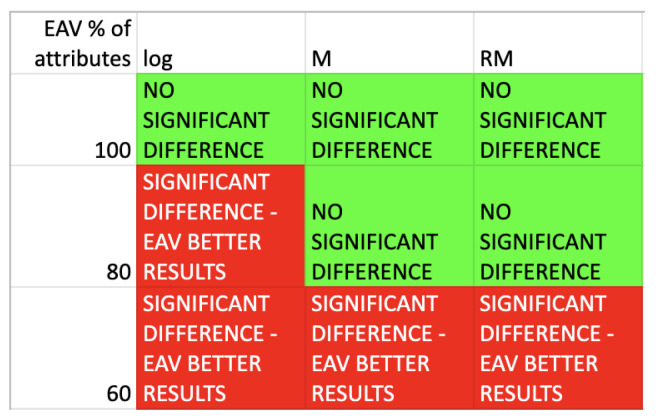
Wilcoxon test results-comparison of the average rules length.

The statistical analysis by means of the Wilcoxon two-tailed test has been performed, to verify the null hypothesis that there are no significant differences in the assessment of rule from the point of view of length and support, average values of these measures have been taken into consideration. The results of rule length comparison have been gathered in the Figure 6.

**Figure 7:**
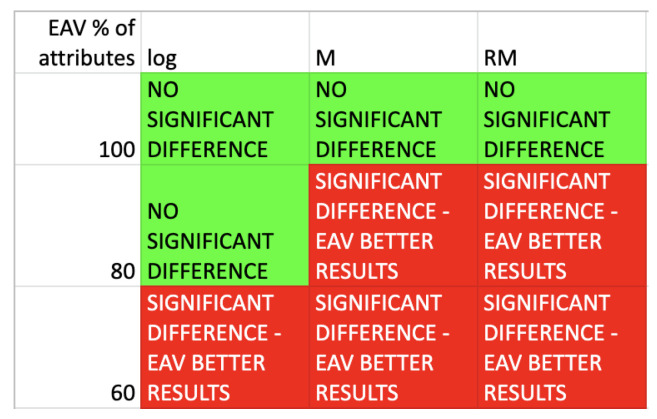
Wilcoxon test results-comparison of the average rules support.

The results of rule support comparison have been gathered in the Figure 7.

The results show that the values of supports are comparable for all heuristics and 100% of attributes for presented algorithm. For 80% and 60% of selected best attributes, the supports results are noticeably better for the proposed approach. As for rule lengths, values are also comparable for all heuristics and 100% of attributes for the presented approach. Taking into account 80% and 60% of selected best attributes, it is possible to see that the length vales are noticeable smaller for the presented algorithm.

### 5.2. Comparison from The Point of Data Classification

From the point of view of classification, accuracy has been compared (see Table 7 and Table 8). 10-fold cross validation has been performed. Column std in presented tables denotes standard deviation of obtained results.

**Figure 8:**
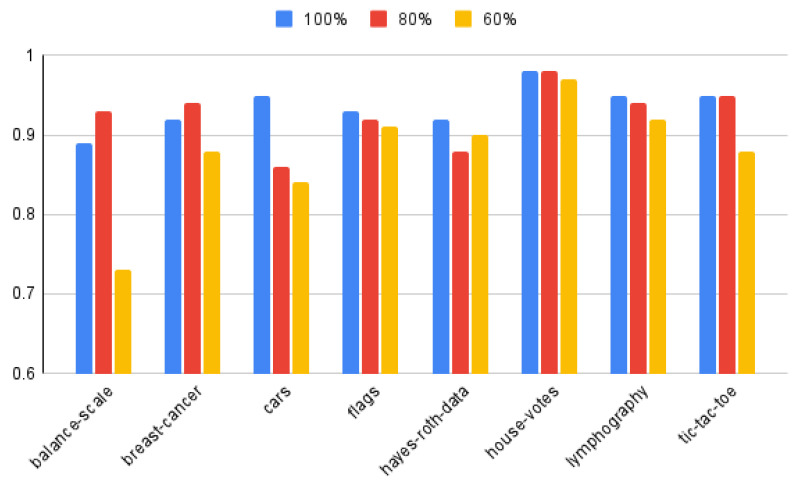
The average accuracy of classification, obtained for 100%, 80% and 60% of best attributes from the ranking.

Figure 8 presents, the average accuracy of classification, obtained for 100%, 80% and 60% of best attributes from the ranking, for considered datasets. For four datasets, i.e., balance-scale, breast-cancer, house-votes and tic-tac-toe, the classification accuracy obtained for 80% of best attributes from the ranking is higher or the same as results obtained for the whole set of attributes.

**Figure 9:**
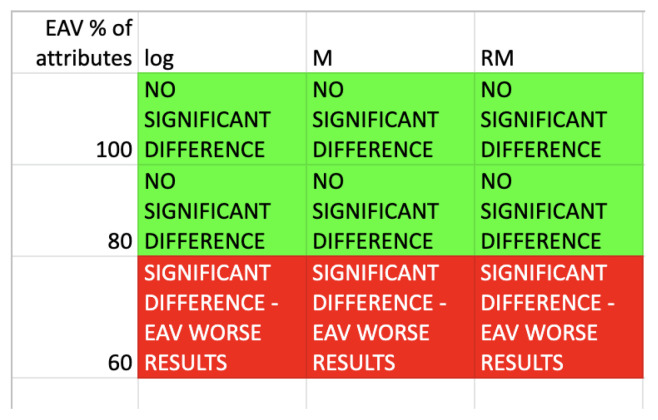
Wilcoxon test results-comparison of the average classification accuracy.

The classification accuracy results once again have been compared by means of two-tailed Wilcoxon test, average values have been taken into this comparison, to verify the null hypothesis that there are no significant differences in the assessment of rule from the point of view of classification accuracy. The results are shown in the Figure 9.

The results show that the classification accuracies are comparable for all heuristics and 100% as well as 80% of selected best attributes for proposed algorithm. For 60% of selected best attributes the classification results are noticeably worse, for the proposed approach. Such a situation is opposite to results obtained from knowledge representation point of view.

## 6. Conclusions

Taking into account results obtained by the experiments performed, it is possible to say that the proposed algorithm allows to obtain rules enough good from both perspectives: data representation and classification. The described approach is a heuristic one, and it has been compared with *M*, RM and log heuristics, which are good from the point of view of knowledge representation. The obtained result show that the presented approach allows to construct rules which are comparable with the heuristics in terms of classification accuracy (except for 60% of selected best attributes). As for rule support and rule length it was shown that the proposed algorithm allows to construct enough short rules with sufficiently good support.

Unfortunately, the proposed algorithm does not allow to automatically perform the feature selection stage. This issue will be considered as the next step on algorithm’s improvement. Additionally, the possibility of working with missing values of attributes will be studied. Future works will also concentrate on comparison with algorithms for induction of decision rules based on sequential covering approach.

## Figures and Tables

**Table 1:** Values on minimum, average and maximum length and support of rules generated by proposed algorithm taking into account the whole set of attributes in data table.

Data Set	Number of		Length			Support		
	Rows	Attributes	Min	Avg	Max	Min	Avg	Max
balance-scale	625	4	3	3.64	4	1	2.44	5
breast-cancer	266	9	1	5.61	9	1	2.61	11
cars	128	6	2	3.90	6	1	79.31	192
flags	194	26	2	8.88	20	1	1.78	6
hayes-roth-data	69	5	1	2.64	4	1	3.81	12
house-votes	279	16	3	6.14	16	1	31.21	81
lymphography	148	18	1	8.40	16	1	2.85	6
tic-tac-toe	958	9	3	5.71	8	1	6.43	38

**Table 2:** Values on minimum, average and maximum length and support of rules generated by proposed algorithm taking into account 80% of best attributes from the ranking.

Data Set	Number of		Length			Support		
	Rows	Attributes	Min	Avg	Max	Min	Avg	Max
balance-scale	625	4	3	3.64	4	1	2.44	5
breast-cancer	266	9	1	5.58	8	1	2.61	11
cars	128	6	2	3.52	5	2	79.88	192
flags	194	26	2	8.88	20	1	1.78	6
hayes-roth-data	69	5	1	2.64	4	1	3.81	12
house-votes	279	16	3	6.09	13	1	31.23	81
lymphography	148	18	1	8.39	15	1	2.85	6
tic-tac-toe	958	9	3	5.71	8	1	6.43	38

**Table 3:** Values on minimum, average and maximum length and support of rules generated by proposed algorithm taking into account 60% of best attributes from the ranking.

Data Set	Number of		Length			Support		
	Rows	Attributes	Min	Avg	Max	Min	Avg	Max
balance-scale	625	4	3	3.00	3	2	3.94	5
breast-cancer	266	9	1	5.28	6	1	3.01	11
cars	128	6	2	3.11	4	6	82.78	192
flags	194	26	2	8.79	16	1	1.78	6
hayes-roth-data	69	5	1	2.51	3	1	3.94	12
house-votes	279	16	3	5.94	10	1	31.46	81
lymphography	148	18	1	8.17	11	1	3.01	6
tic-tac-toe	958	9	3	5.29	6	1	6.73	38

**Table 4:** Values on minimum, average and maximum length and support of rules generated by means of *M* heuristic.

Data Set	Number of		Length			Support		
	Rows	Attributes	Min	Avg	Max	Min	Avg	Max
balance-scale	625	4	3	3.41	4	1	3.38	5
breast-cancer	266	9	1	2.97	6	1	2.81	24
cars	128	6	1	5.57	6	1	6.69	576
flags	194	26	1	2.04	4	1	2.04	18
hayes-roth-data	69	5	1	2.88	4	1	2.33	12
house-votes	279	16	2	3.17	6	1	22.86	95
lymphography	148	18	1	2.32	4	1	5.34	32
tic-tac-toe	958	9	3	4.12	5	1	7.32	90

**Table 5:** Values on minimum, average and maximum length and support of rules generated by means of RM heuristic.

Data Set	Number of		Length			Support		
	Rows	Attributes	Min	Avg	Max	Min	Avg	Max
balance-scale	625	4	3	3.41	4	1	3.38	5
breast-cancer	266	9	1	3.52	8	1	3.25	24
cars	128	6	1	5.44	6	1	8.14	576
flags	194	26	1	2.23	9	1	2.59	18
hayes-roth-data	69	5	1	2.92	4	1	2.56	12
house-votes	279	16	2	3.29	5	1	32.22	95
lymphography	148	18	1	2.56	5	1	7.70	32
tic-tac-toe	958	9	3	4.32	7	1	13.21	90

**Table 6:** Values on minimum, average and maximum length and support of rules generated by means of log heuristic.

Data Set	Number of		Length			Support		
	Rows	Attributes	Min	Avg	Max	Min	Avg	Max
balance-scale	625	4	3	3.41	4	1	3.38	5
breast-cancer	266	9	1	3.29	6	1	4.10	25
cars	128	6	1	5.45	6	1	8.11	576
flags	194	26	1	3.26	6	1	5.68	22
hayes-roth-data	69	5	1	2.90	4	1	2.87	12
house-votes	279	16	2	3.56	7	2	40.02	95
lymphography	148	18	1	2.85	5	1	10.83	32
tic-tac-toe	958	9	3	4.20	6	2	13.04	90

**Table 7:** Average classification accuracies of rules generated by means of the proposed algorithm.

Data Set	100%	Std	80%	Std	60%	Std
balance-scale	0.89	0.05	0.93	0.05	0.73	0.05
breast-cancer	0.92	0.03	0.94	0.03	0.88	0.03
cars	0.95	0.06	0.86	0.04	0.84	0.07
flags	0.93	0.05	0.92	0.05	0.91	0.05
hayes-roth-data	0.92	0.07	0.88	0.07	0.90	0.05
house-votes	0.98	0.03	0.98	0.03	0.97	0.04
lymphography	0.95	0.11	0.94	0.11	0.92	0.04
tic-tac-toe	0.95	0.06	0.95	0.06	0.88	0.06

**Table 8:** Average classification accuracies of rules generated by means of M, RM and log heuristics.

Data Set	M	Std	RM	Std	Log	Std
balance-scale	0.94	0.06	0.95	0.05	0.95	0.05
breast-cancer	0.94	0.03	0.95	0.03	0.95	0.03
cars	0.97	0.11	0.97	0.11	0.97	0.11
flags	0.97	0.08	0.99	0.08	0.99	0.08
hayes-roth-data	0.94	0.07	0.94	0.07	0.94	0.07
house-votes	0.99	0.11	0.99	0.11	0.99	0.11
lymphography	0.94	0.05	0.98	0.06	0.98	0.06
tic-tac-toe	0.97	0.04	0.98	0.05	0.98	0.05

## Data Availability

Publicly available datasets were analyzed in this study. This data can be found here: http://archive.ics.uci.edu/ml (accessed on 23 March 2022).
